# Supplementary material for: Comparison of high-throughput single-cell RNA-seq methods for ex vivo drug screening
Source: NAR Genom Bioinform. 2024 Jan 29;6(1):lqae001. doi: 10.1093/nargab/lqae001 (PMC10823582; doi:10.1093/nargab/lqae001)
Supplement: lqae001_Supplemental_Files [file Gezelius_Supplmentary_Figures_R2.pdf › Gezelius_Supplmentary_Figures_R2.pdf]

COMPARISON OF HIGH-THROUGHPUT SINGLE-CELL RNA-SEQ METHODS FOR EX VIVO DRUG SCREENING

AUTHORS

Henrik Gezelius[1], Anna Pia Enblad[1,2], Anders Lundmark[1], Martin Åberg[1,3], Kristin Blom [1,3], Jakob Rudfeldt [1,3], Amanda Raine[1], Arja Harila[2], Verónica Rendo[4], Merja Heinäniemi[5], Claes Andersson[1,3], Jessica Nordlund[1]*

[1] Department of Medical Sciences and Science for Life Laboratory, Uppsala University, Uppsala, 751 44, Sweden

[2] Department of Women's and Children's Health, Uppsala University, Uppsala, 751 85, Sweden

[3] Department of Clinical Chemistry and Pharmacology, Uppsala University Hospital, Uppsala, 751 85, Sweden

[4] Department of Immunology, Genetics and Pathology, Uppsala University, Uppsala, 751 85, Sweden

[5] School of Medicine, University of Eastern Finland, 70210 Kuopio, Finland

* To whom correspondence should be addressed. Tel: +46 70-4250806; Email: jessica.nordlund@medsci.uu.se

Figure S1

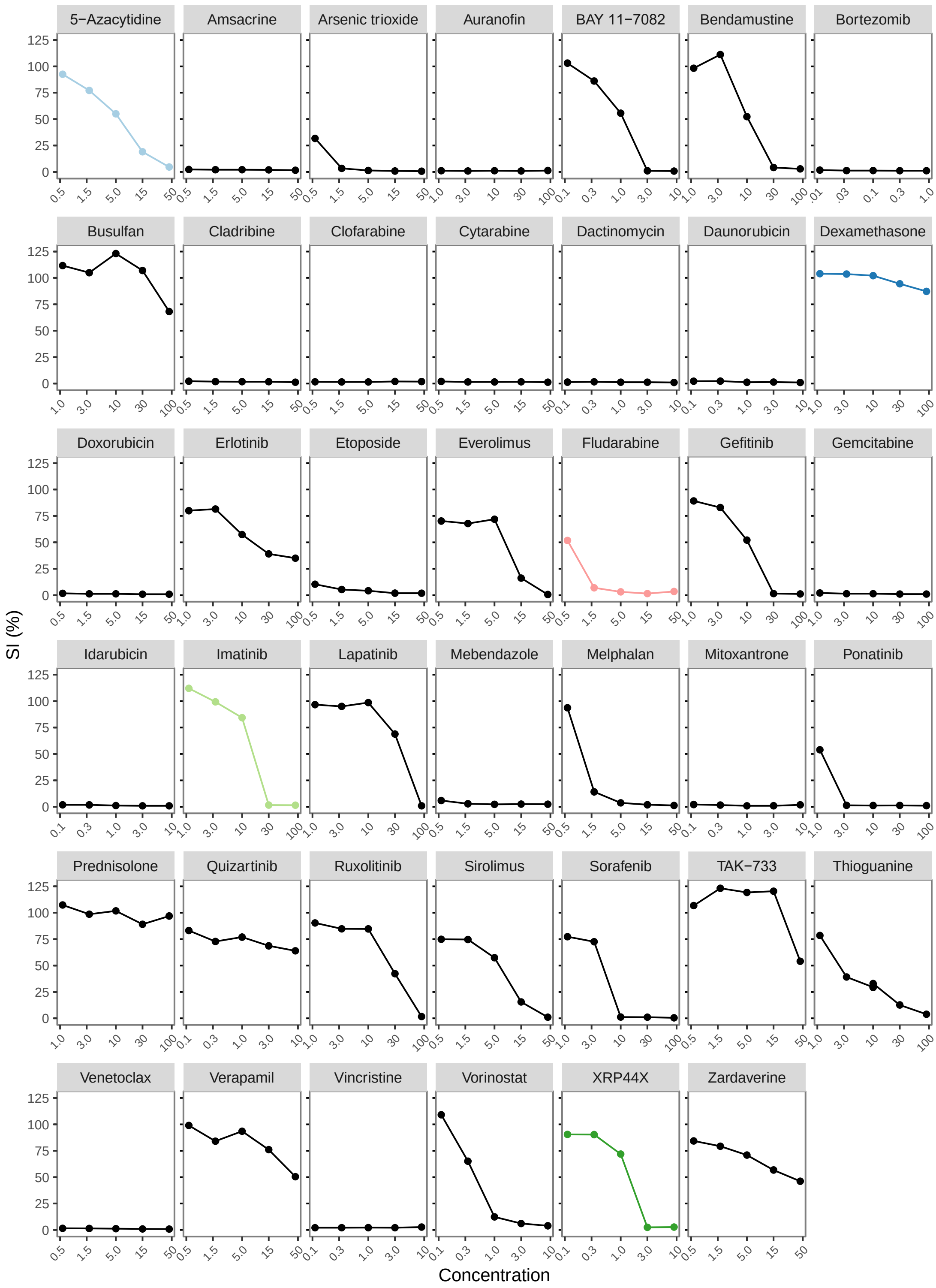

Supplementary Figure S1. Survival index of Reh cells exposed to 72 hours treatment of cytostatic treatment at the concentrations as indicated in each panel. Each dot represents the mean measured from two replicates. The cells surviving the following treatments were selected for single-cell RNA-seq: 5-Azacytidine (light blue), Dexamethasone (dark blue), Fludarabine (pink), Imatinib (light green), and XRP44X (dark green).

Figure S2

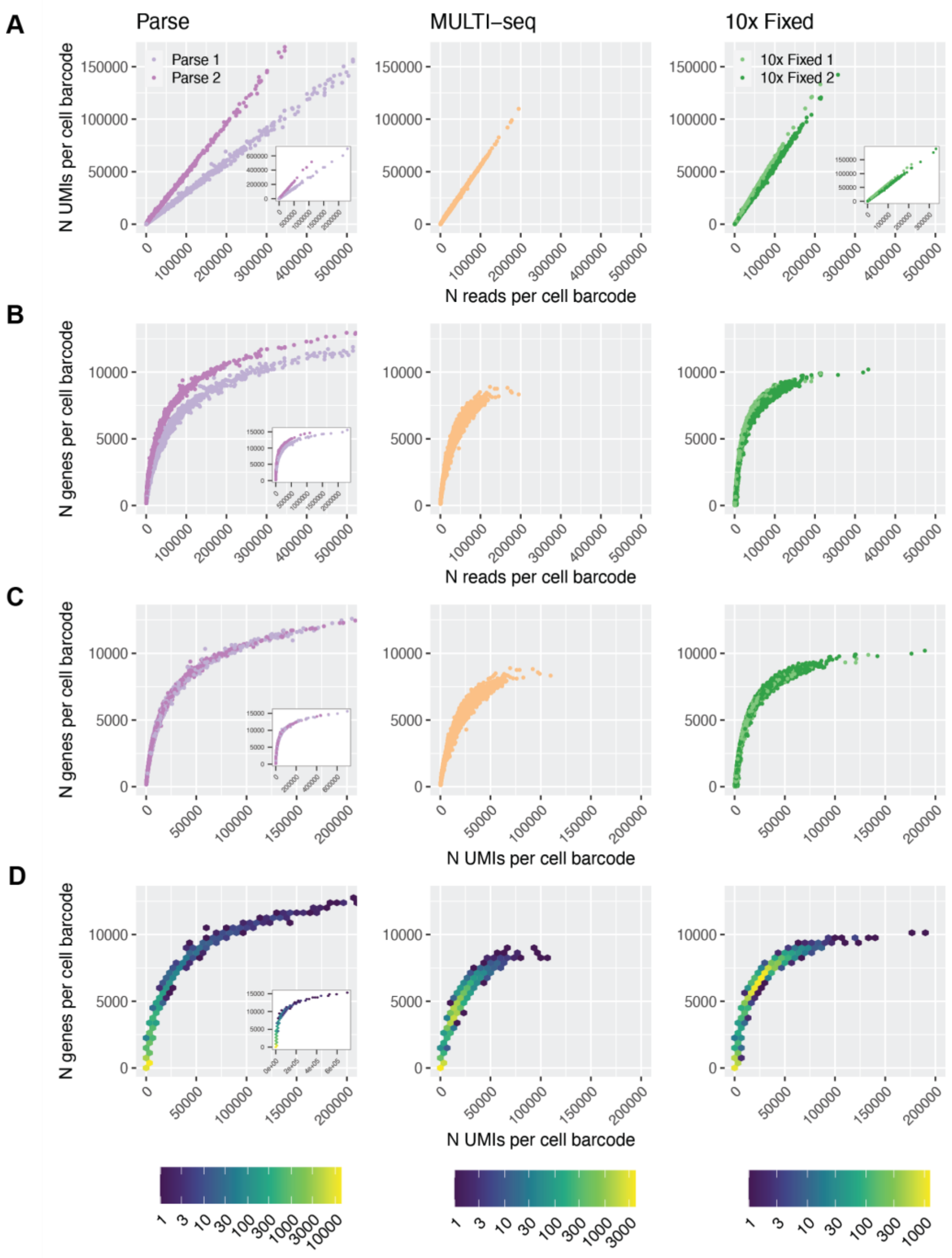

Supplementary Figure S2. Comparisons of sequencing depth, UMIs and number of genes detected prior to cell filtering for each scRNA-seq library method. All values are presented per cell barcode. (A) The number of reads (x-axis) plotted against the number of UMIs (y-axis). Note that each sub-library has a linear correlation, while in the sub-libraries with different read depth the correlation factor is not equal. The inset for the Parse data shows the full range of barcodes. (B) The number of genes detected plotted against number. (C) The number of genes detected plotted against number of UMIs. (D) The number of genes detected plotted against the number of UMIs detected visualized as hexagonal binning plot, the color indicates the number of cell-barcodes contributing to each hexagonal bin according to each scale below each panel.

Figure S3

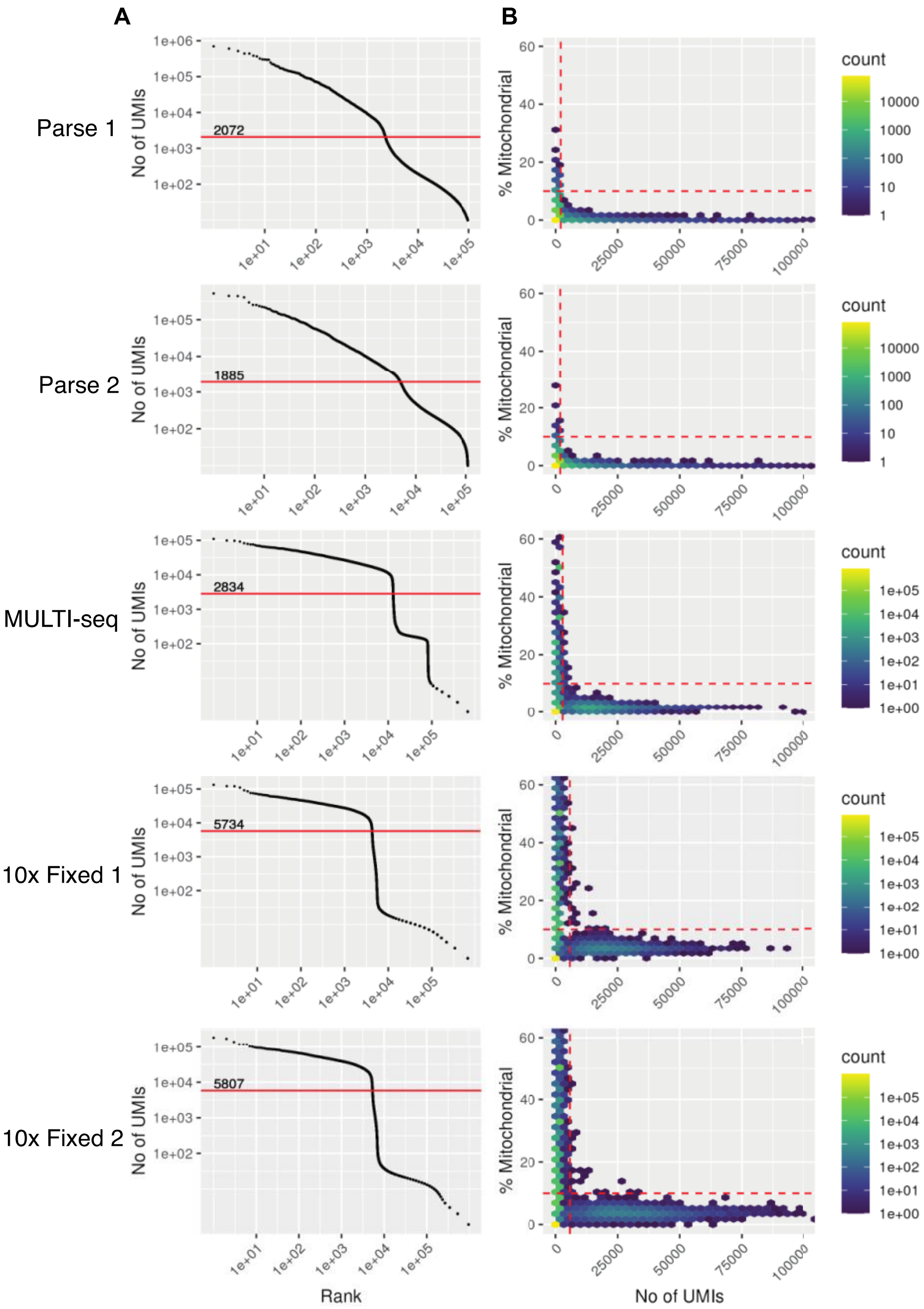

Supplementary Figure S3. Filtering selection for removing non-cell UMIs. (A) Knee bar plots for each sequencing library, the y-axis indicates the number of UMIs and the x-axis the rank of each index. The library (sub-library) is indicated to the left of the panel. The red line indicates the inflection point, only barcodes with detected UMIs above the given number are considered to be cells used for downstream analyses. (B) Histogram of the percent of reads mapping to mitochondrial genes (y-axis) and the number of UMIs (x-axis), visualized as hexagonal binning plots. The color indicates the number of cells contributing to each hexagonal bin according the scale to the right of each panel. The dashed red vertical line indicates the cut-off of the inflection point from the corresponding plot to the left (panel A). The dashed red horizontal line indicates the cut-off for filtering out cells with >10% sequencing reads that map to mitochondrial genes.

Figure S4

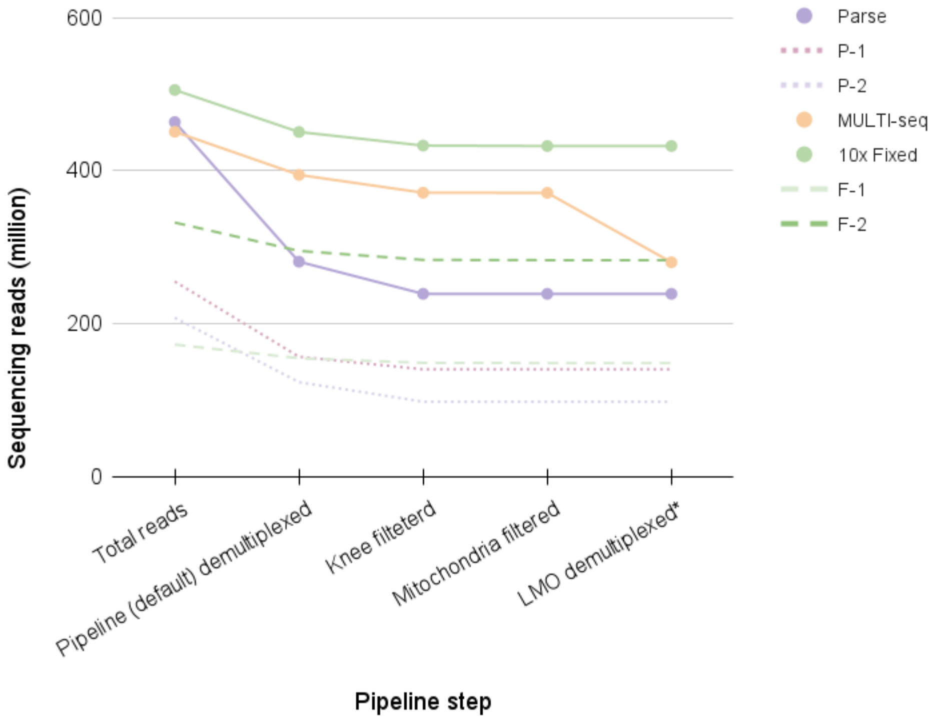

Supplementary Figure S4. Reads remaining during the data processing pipeline. The number of reads retained at each stage of the pipeline is delineated by method (Parse, MULTI-seq, 10x Fixed) and sub-library (P-1, P-2, F-1, and F-2) as indicated in the legend to the right of the panel. Notably, the lipid-modified oligo (LMO) demultiplexing step (LMO demultiplexed*) was exclusively conducted for the MULTI-seq library.

Figure S5

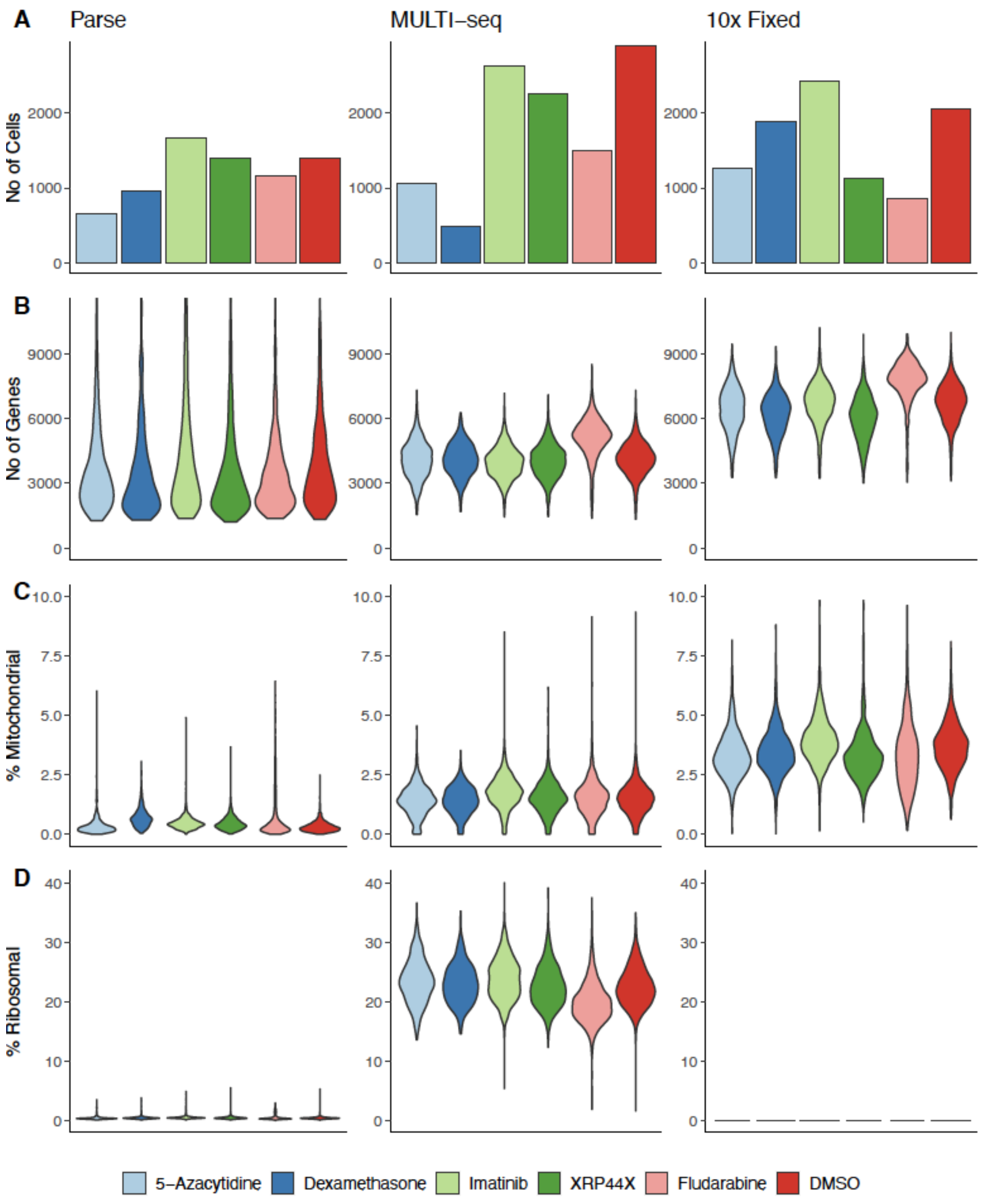

Supplementary Figure S5. Quality metrics of single cell RNA sequencing (scRNA-seq) data generated from three different library preparation methods (Parse, MULTI-seq, 10x Fixed) separated by drug condition (barcode). The color key for the drug conditions in indicated at the bottom of the figure. (A) The number of cells detected by each scRNA-seq library preparation method and condition presented as bar plots. (B) The number of genes detected by each scRNA-seq library preparation method and condition presented as violin plots. (C) The percentage of reads that map to mitochondrial genes by scRNA-seq library preparation method and condition presented as violin plots. (D) The percentage reads that map to ribosomal genes by scRNA-seq library preparation method and condition presented as violin plots.

Figure S6

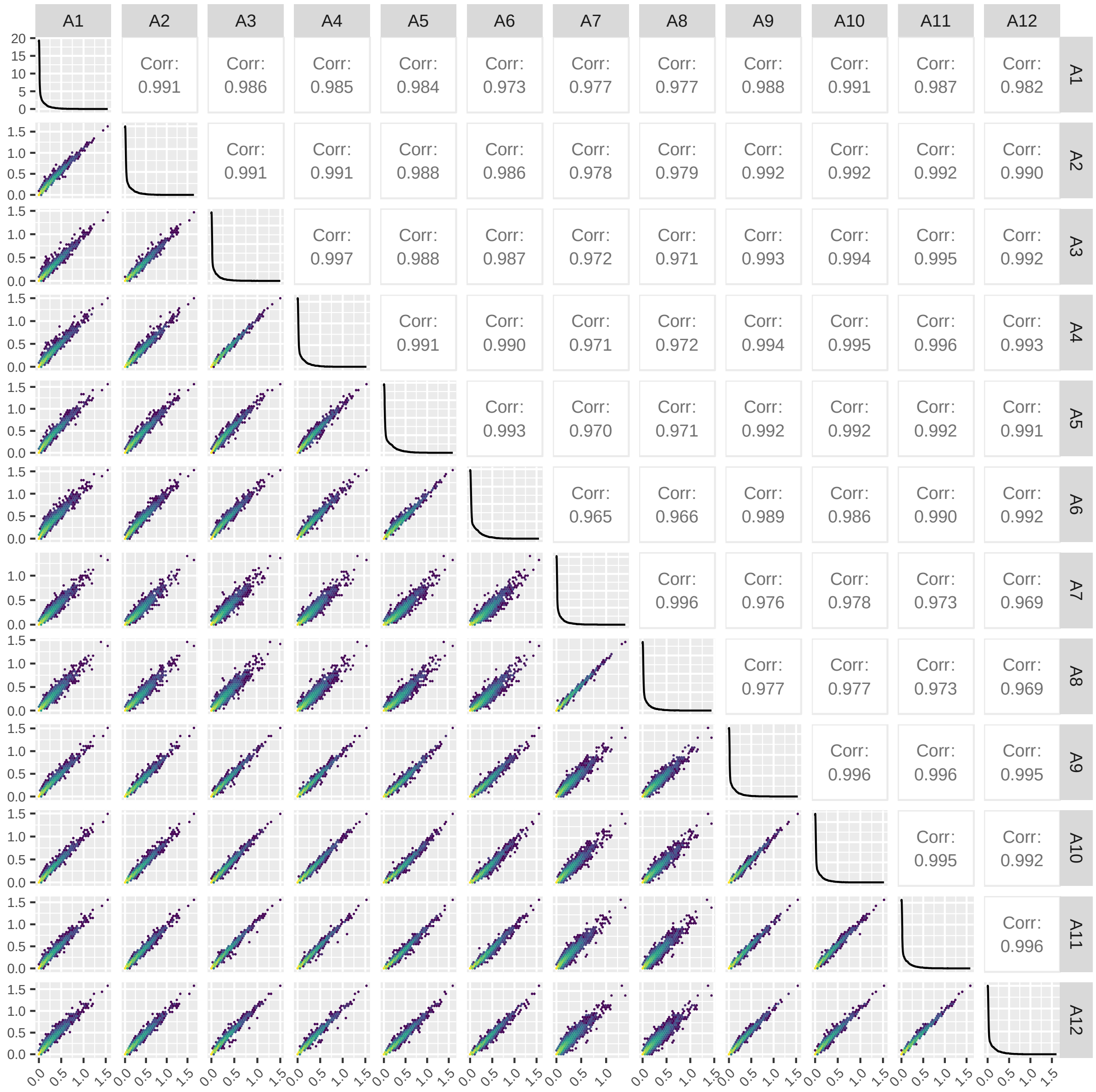

Supplementary Figure S6: Pairwise correlation of Parse sample wells. Each panel contains pairwise log-transformed pseudo-bulk expression values for the indicated sample wells. The correlation values are plotted in the opposite intersection. Density plots along the diagonal indicate the distribution of expression values within the indicated well. Wells A1-A2 correspond to 5-azacytidine treated cells, A3-A4 dexamethasone, A5-A6 imatinib, A7-A8 fludarabine, A9-A10 XRP44X, and A11-A12 control (DMSO).

Gezelius et al Supplementary Figures

Figure S7

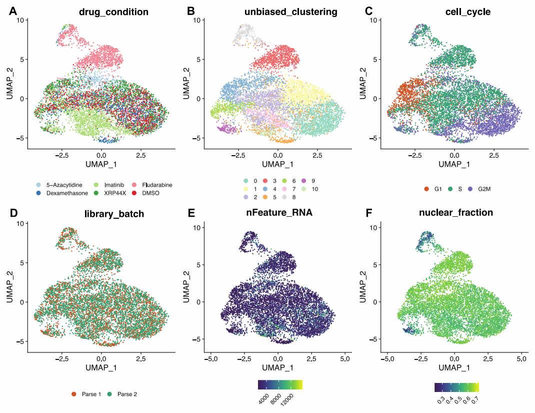

Supplementary Figure S7. UMAP visualization of Parse single-cell RNA-seq data. The cells are color-coded to depict various biological attributes: (A) Drug condition/barcode, (B) Unbiased clustering using FindClusters in the R package "Seurat", (C) Cell cycle estimation, (D) Sub-library, (E) Number of genes detected, and (F) Fraction of nuclear reads, calculated as N intronic reads / (N intronic reads + N exonic reads) using the R package "DropletQC".

Gezelius et al Supplementary Figures

Figure S8

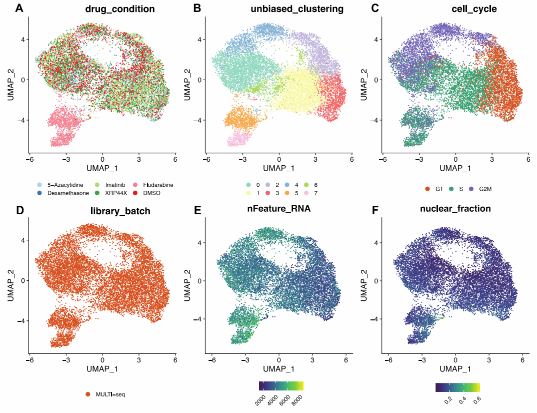

Supplementary Figure S8. UMAP visualization of MULTI-seq single-cell RNA-seq data. The cells are color-coded to depict various biological attributes: (A) Drug condition/barcode, (B) Unbiased clustering using FindClusters in the R package "Seurat", (C) Cell cycle estimation, (D) library batch (for MULTI-seq there was only one batch), (E) Number of genes detected, and (F) Fraction of nuclear reads, calculated as N intronic reads / (N intronic reads + N exonic reads) using the R package "DropletQC".

Gezelius et al Supplementary Figures

Figure S9

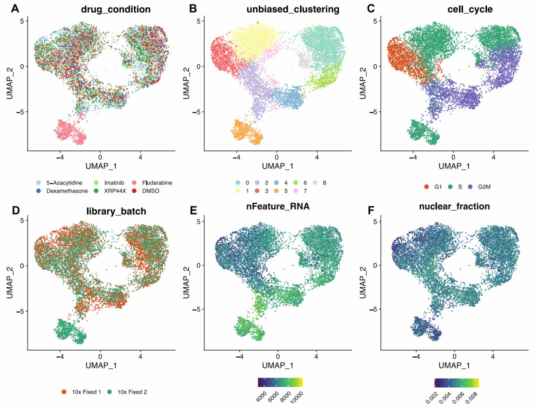

Supplementary Figure S9. UMAP visualization of 10x Fixed single-cell RNA-seq data. The cells are color-coded to depict various biological attributes: (A) Drug condition/barcode, (B) Unbiased clustering using FindClusters in the R package "Seurat", (C) Cell cycle estimation, (D) Library preparation batch, (E) Number of genes detected, and (F) Fraction of nuclear reads, calculated as N intronic reads / (N intronic reads + N exonic reads) using the R package "DropletQC".

Figure S10

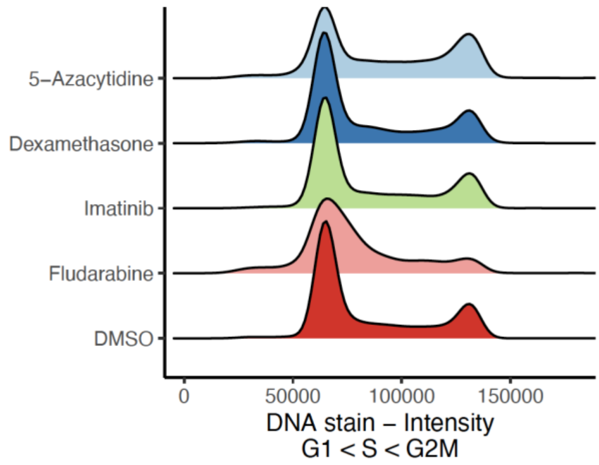

Supplementary Figure S10. Cell cycle states after 72 hours of treatment. Ridge plot with population histogram with the DNA content measured by DAPI intensity (x-axis), the density of cells for each treatment is represented on the y-axis.
